# Reassessing intertemporal choice: human decision-making is more optimal in a foraging task than in a self-control task

**DOI:** 10.3389/fpsyg.2015.00095

**Published:** 2015-02-06

**Authors:** Evan C. Carter, Eric J. Pedersen, Michael E. McCullough

**Affiliations:** ^1^Department of Psychology, University of MiamiCoral Gables, FL, USA; ^2^Department of Ecology, Evolution and Behavior, University of MinnesotaSaint Paul, MN, USA

**Keywords:** intertemporal choice, impulsive choice, delay discounting, self-control, impulsivity, decision making, foraging

## Abstract

Many contemporary concerns (e.g., addiction, failure to save) can be viewed as intertemporal choice problems in which the consequences of choices are realized at different times. In some laboratory paradigms used to study intertemporal choice, non-human animals demonstrate a preference for immediacy (impulsive choice) that results in failures to maximize the amount of reward received. There is evidence, however, suggesting that such non-optimal impulsive choice may be due to a mismatch between the standard presentation of options in the laboratory (e.g., a “larger-later” and a “smaller-sooner” option) and the way that options occur in natural settings (e.g., foraging). We present evidence that human impulsive choice is similarly affected: in two experiments, decisions were more optimal when options were presented in a format sharing features with the evolutionarily important problem of foraging compared to when options were presented in the standard format. These findings suggest a more nuanced view of intertemporal choice and support the adoption of ideas from foraging theory into the study of human decision making.

## INTRODUCTION

**FIGURE 1:**
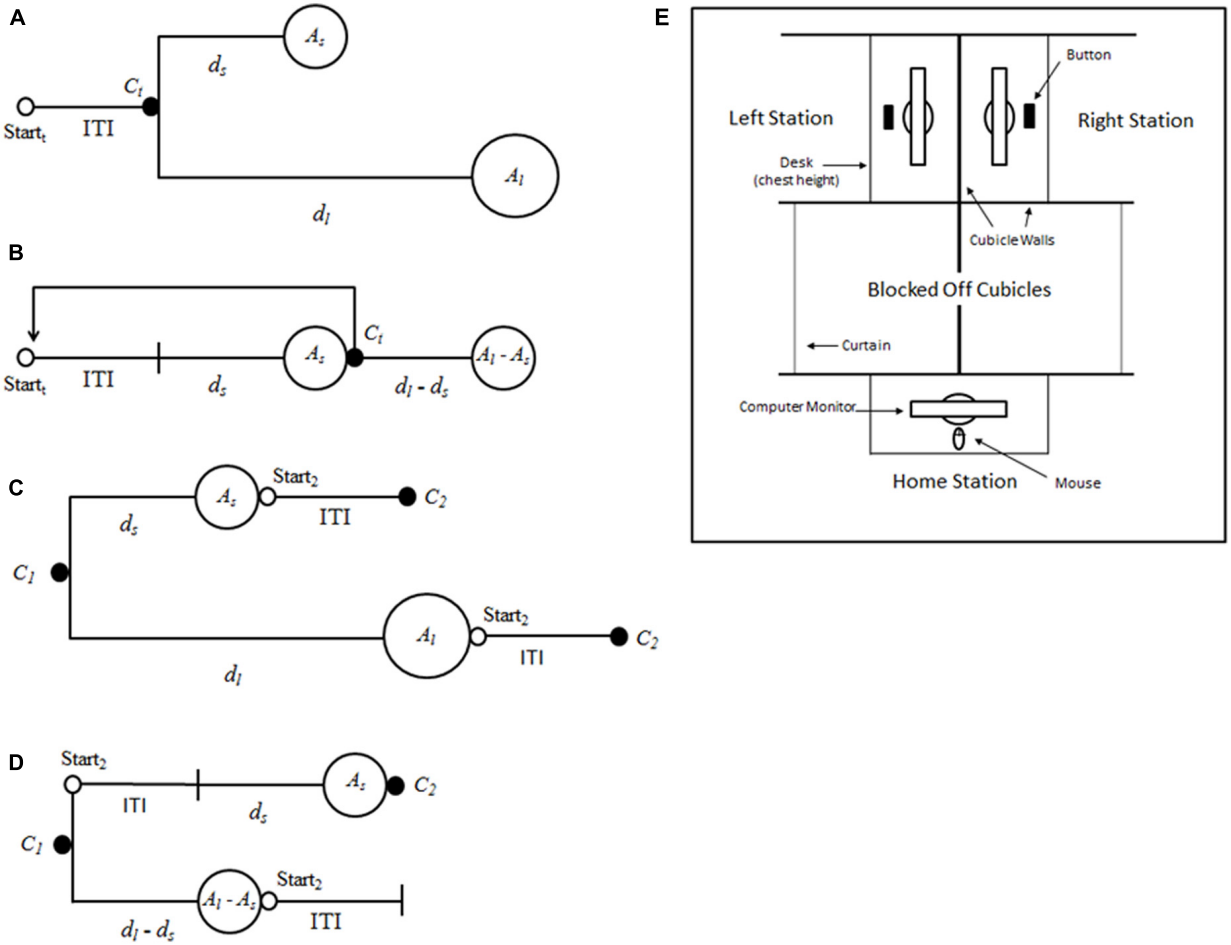
**Schematics for the self-control and patch paradigms. (A)** Self-control: trial *t* begins at Start_t_. When the participant reaches the choice point, *C_t_*, he or she must decide between amount *A_l_* (after a delay of *d_l_*) and amount *A_s_* (after a delay of *d_s_*). **(B)** Patch: the participant waits for the duration of the inter-trial interval (ITI), then waits for the duration of *d_s_* and receives *A_s_*. At *C_t_* the participant chooses to either receive *A_l_*–*A_s_* after a delay of *d_l_*–*d_s_*, or begin a new trial. **(C)** The self-control and **(D)** the patch paradigm viewed from the first choice point, *C_*1*_*. Note that at *C_*1*_* in patch, the total delay to *A_s_* includes the ITI for the second trial, whereas the ITI has already passed when a subject reaches *C_*1*_* in the self-control paradigm. **(E)** Schematic representing bird’s-eye view of the laboratory set-up in experiment one.

Important social concerns, such as addiction, obesity, and debt, can be conceptualized as intertemporal choice problems—choices between consequences that are realized differently through time (Madden and Bickel, 2010). Intertemporal choice is frequently studied by having subjects choose between two mutually exclusive reward, where the smaller reward is available sooner and the larger reward is available later (**Figure 1A**), a task that has been called *the self-control paradigm* (Logue, 1988). To varying degrees, human and non-human animals in the self-control paradigm show a preference for smaller-sooner reward, called impulsive choice, even when such preference is non-optimal in that it decreases the total amount of reward received (Madden and Bickel, 2010). Here, we demonstrate that human intertemporal choice appears to be at least partially due to the manner in which options are presented in laboratory tasks. Additionally, we argue that researchers should consider a broader view of intertemporal choice—one that takes into account choice problems other than the larger-later/smaller-sooner presentation of the self-control paradigm.

### INTERTEMPORAL CHOICE BY NON-HUMAN ANIMALS

Experiments on non-human animals typically find that delaying reward causes them to lose much of their value, a phenomenon referred to as *temporal* or *delay discounting* (Madden and Bickel, 2010). For example, Stephens (2002) estimated that the first second of delay caused food rewards to lose 75% of their value for pigeons in the classic self-control paradigm experiment by Mazur (1987). Additionally, the proportion of choice for the larger-later reward relative to the smaller-sooner reward is commonly quite low. For example, Tobin and Logue (1994) reviewed three experiments—one using rats (Tobin et al., 1993) and two using pigeons (Mazur and Logue, 1978; Logue et al., 1985) and showed that, on average, rats and pigeons picked the larger-later option only 20% and 3% of the time, respectively, despite the fact that it provided three times more food than the smaller-sooner option. Furthermore, non-human animals’ choices in the self-control paradigm seem to be determined almost entirely by the delay until receipt of a reward (i.e., *the prereinforcer delay*), rather than other timing components, such as delay following receipt of reward (i.e., *postreinforcer delay*; e.g., Logue et al., 1985; Mazur et al., 1985). As a result, choice of the smaller-sooner option is usually non-optimal in the sense that it produces less food over the course of a session than the larger-later option. For example, Logue et al. (1985) found that impulsive choice by pigeons resulted in receipt of only 34.6% of the food that could have been obtained. Such findings are often interpreted as showing that non-human animals lack self-control—they forego the benefits of delaying gratification by impulsively choosing the more immediately available reward.

Importantly, the self-control paradigm is only one of the ways in which researchers have studied intertemporal choice by non-human subjects. Animals’ foraging behavior in nature also involves choosing between options that differ in value and in time until consumption, and biologists have long studied such intertemporal choice from the perspective of Optimal Foraging Theory (Stephens and Krebs, 1986). For example, a starling foraging for food in a plot of grass faces the choice of continuing to spend time searching in that location or to spend time flying to a new location that might offer better or more food. This example can be analyzed using the patch model (Charnov, 1976; Stephens and Krebs, 1986): in an environment where food is more densely distributed in particular locations, or “patches,” a forager will inevitably experience diminishing returns as food in the occupied patch is depleted. Thus, the optimal decision-making strategy is determined by comparing the present rate of intake in the patch to the rate of intake that can be expected elsewhere in the environment, and to leave the patch when doing so is likely to result in achieving a higher rate of gain [i.e., maximization of long-term rate (LTR) of food obtained during a foraging bout]. Although rate maximization models like the patch model often fail to completely explain observed behavior, researchers tend to find that animals are sensitive to LTR and that behavior is at least in qualitative agreement with model predictions (Stephens and Krebs, 1986; Nonacs, 2001).

Given that choice during foraging and choice in the self-control paradigm are both forms of intertemporal choice, an important issue is why the assumption of LTR maximization, which has served well when predicting behavior in the foraging literature, has consistently fared less well when predicting behavior in the self-control paradigm? To investigate this discrepancy, Stephens and Anderson (2001) designed *the patch paradigm* (**Figure 1B**), which reframes the larger-later/smaller-sooner choice of the self-control paradigm as a leave/stay choice, akin to that which animals must make when foraging in patches. They compared the choices of blue jays in the two paradigms for which delays to the small and large reward (*d_s_* and *d_l_*), amounts of the small and large reward (*A_s_* and *A_l_*), and the duration of intertrial interval (ITI) were set so that the LTRs for each option, $\frac{A_{s}}{ITI+d_{s}}$ and $\frac{A_{l}}{ITI+d_{l}}$were equal across paradigms.

A simple LTR-maximizing rule can be formulated based on the difference between the LTRs of two options: let ΔLTR be the change in LTR when *A_l_* is picked instead of *A_s_*, such that

$$\Delta LTR=\frac{A_{l}}{ITI+d_{l}}-\frac{A_{s}}{ITI+d_{s}}$$

When ΔLTR > 0, a choice of *A_l_* maximizes LTR, but when ΔLTR < 0, a choice of *A_s_* maximizes LTR. When ΔLTR = 0, neither option is superior in terms of LTR. Stephens and Anderson (2001) found that the jays made decisions that were more consistent with the ΔLTR rule when in the patch paradigm compared to in the self-control paradigm—that is, the jays’ decision-making was more optimal in the patch paradigm than in the self-control paradigm—a pattern of results we will refer to as *the patch effect*.

Stephens and Anderson’s (2001) results led them to formulate the ecological rationality hypothesis of impulsivity (see also Stevens and Stephens, 2010), which is that the impulsivity observed in the self-control paradigm is due to the fact that the self-control paradigm is a poor model of the natural choice problems that organisms have evolved to solve. Specifically, they proposed that animals have evolved to make decisions based on an assessment of only the time until the next reward [i.e., short-term rate (STR) maximization], and they observed that when a decision rule akin to the ΔLTR rule above is formulated for STR, following the ΔSTR rule results in choosing the same option as following the ΔLTR rule in the patch paradigm, but can result in a violation of the ΔLTR rule in the self-control paradigm. This occurs because, in the patch paradigm, every delay follows a choice and ends in reward receipt, whereas in the self-control paradigm, the ITI occurs after receipt of reward and before choice, and so is ignored in the calculation of STR (**Figures 1C,D**). The proposal that non-human animals use a STR-based rule is supported by the finding that they mostly ignore postreinforcer delays (e.g., Logue et al., 1985; Mazur et al., 1985), as well as the descriptive power of delay discounting models that hold that discounted value is a function of only the prereinforcer delay (e.g., the hyperbolic model; Mazur, 1987). Moreover, findings from several recent experiments suggest that delay discounting by monkeys is in fact attributable to the under-estimation of the postreinforcer buffer, a type of postreinforcer delay that, in some versions of the self-control paradigm, follows choice of the smaller-sooner reward (Pearson et al., 2010; Blanchard et al., 2013).

Subsequent direct tests (using blue jays) have shown that subjects do not purely use STR-maximization strategies, thereby making such an explanation of the patch effect unlikely; however, some methodological explanations for the patch effect have been ruled out, and similar results to those of Stephens and Anderson (2001) have been reported (Stephens and McLinn, 2003; Stephens and Dunlap, 2009). Additionally, the behavioral difference between paradigms is indirectly supported by other experiments in which the behavior of subjects of the same species is poorly explained by LTR-maximization when observed in the self-control paradigm, but well-explained by such a strategy when observed in foraging tasks: for example, Bateson and Kacelnik (1996) found that starlings’ choices were better explained as STR-maximization than as LTR-maximization in three experiments using the self-control paradigm, but different individuals from the same species have been found to behave approximately optimally in patch-foraging experiments (Kacelnik, 1984; Cuthill, 1985). Likewise, rats have been found to choose impulsively in the self-control paradigm (e.g., Tobin et al., 1993), but optimally under some conditions in a patch-foraging task (Mellgren et al., 1984). The implication of these findings is that intertemporal choice varies as a function of the specific problem being faced, and that a fully developed theory of intertemporal choice must take into account more than just the self-control paradigm.

### EXAMINING HUMAN BEHAVIOR IN THE PATCH PARADIGM

There is an extensive literature documenting correlations between human intertemporal choice in the self-control paradigm (usually operationally defined as a discounting rate) and outcomes such as addiction, pathological gambling, and the presence of psychiatric disorders (Madden and Bickel, 2010). Indeed, some have argued that overly steep delay discounting should be considered a trans-disease process (i.e., a process underlying multiple physical, mental, and behavioral disorders; Bickel et al., 2012), and researchers have begun investigating ways to modify delay discounting (e.g., Koffarnus et al., 2013). Furthermore, much work has been done toward elucidating the neural mechanisms that mediate choice in the self-control paradigm (Peters and Büchel, 2011).

Given the amount of interest in human intertemporal choice, an obvious question is whether humans, like blue jays, behave more optimally in the patch paradigm than in the self-control paradigm. If so, this finding would be important for the interpretation of results from experiments using the self-control paradigm, since it would suggest that non-optimal behavior by an individual in the self-control paradigm does not necessarily mean that that individual behaves the same way during *all* forms of intertemporal choice. Furthermore, understanding the cause of the patch effect would help researchers better understand the nature of intertemporal choice in the self-control paradigm, which will ultimately be necessary for a complete understanding of why steeper rates of discounting might be a trans-disease process.

The goal of the present experiments was to determine whether humans’ choices were more optimal in the patch paradigm as compared to the self-control paradigm—that is, to test the patch effect. To do this, we adopted the procedures of Stephens and Anderson (2001) for use with human subjects. Previous work has found that in versions of the self-control paradigm that were similar to ours, participants tended to make decisions that were broadly consistent with the use of a LTR-maximizing strategy (e.g., Logue et al., 1986; Flora and Pavlik, 1992; Ito and Nakamura, 1998). For example, in Logue et al.’s (1986) Experiment 1, participants chose the LTR maximizing option 61% of the time on average, and participants in such experiments have reported consciously trying to maximize LTR (Logue, 1988). Therefore, we predicted that participants would generally make choices that were consistent with the ΔLTR rule, regardless of paradigm; however, we also predicted that we would find evidence for the patch effect in that participants’ decisions would be better predicted by the ΔLTR rule in the patch paradigm compared to in the self-control paradigm.

We tested both of these predictions in two experiments. Experiment 1 was based on the procedure Stephens and Anderson (2001) used with blue jays inasmuch as our human participants needed to move between different locations to make their choices. In other words, this design involved spatially distinct patches, a feature shared by all previous experiments testing the patch effect. The procedure in Experiment 2 was identical to that of Experiment 1, except that all trials were completed at a single computer by a seated participant, and rewards were not delivered at spatially distinct locations. This modification was important because it more closely resembled methods that are commonly used in research on choice by humans in the self-control paradigm (e.g., Logue et al., 1986), and it enabled us to evaluate whether the results from Experiment 1 were caused solely by the fact that patches were spatially distinct rather than by a substantive difference between the patch and self-control paradigms.

## MATERIALS AND METHODS

### SUBJECTS

Participants [Experiment 1: *N* = 216 (79 male, 134 female, 3 unreported); Experiment 2: *N* = 163 (88 male, 75 female)] were undergraduate Introduction to Psychology students at the University of Miami who completed sessions for partial course credit and were paid the amount they earned from their choices (see below).

### PROCEDURE

Sessions were completed with one participant at a time for Experiment 1 and up to four participants at a time (in separate carrels) for Experiment 2. Experimental sessions were scheduled for 90-minute blocks, and though participants were told that sessions would take the full 90 minutes, the time spent depended on the programmed delays for each condition.

In all cases, the large reward (*A_l_*) was worth $0.50 and the small reward (*A_s_*) was worth $0.25. The ratio of the magnitudes of *A_l_* and *A_s_* was chosen to match the ratio used by Stephens and Anderson (2001), whereas the magnitudes themselves were chosen because they are comparable to those in pervious experiments using similar procedures (e.g., Flora and Pavlik, 1992) and because they allowed us to represent the reward in terms of U.S. quarter dollar coins (see Supplemental Materials), which we guessed would make them easier for participants to conceptualize as compared to, for example, $0.15.

For both experiments, participants were randomly assigned to one of eight possible combinations of durations for ITI (30 or 90 s), delay to the small reward (*d_s_*: 5 or 50 s), and delay to the large reward (*d_l_*: 60 or 90 s). These delays were taken from those used by Stephens and Anderson (2001). Choosing the large reward instead of the small reward resulted in varying degrees of ΔLTR, depending on the specific combination of durations (**Table 1**). Paradigm order and color (see below) were counter-balanced.

**Table 1:** Short- and long-term rates for choosing *A_**s**_* and *A_**l**_* in the self-control and patch paradigms for all combinations of duration assignments (ITI, *ds*, and *d_**l**_*).

Duration assignments			Short-term rates (STR) for choosing *A_s_* and *A_l_* in self-control			STRs for choosing *A_s_* and *A_l_* in patch			Long-term rates (LTRs) for *A_s_* and *A_l_*		
ITI	*d_s_*	*d_l_*	*A_s_*	*A_l_*	ΔSTR	*A_s_*	*A_l_*	ΔSTR	*A_s_*	*A_l_*	ΔLTR
0.5	0.083	1	301.2	50	-251.2	42.88	27.26	-15.62	42.88	33.33	-9.55
1.5	0.083	1	301.2	50	-251.2	15.79	27.26	11.47	15.79	20	4.21
0.5	0.83	1	30.12	50	19.88	18.8	147.06	128.26	18.8	33.33	14.54
1.5	0.83	1	30.12	50	19.88	10.73	147.06	136.33	10.73	20	9.27
0.5	0.083	1.5	301.2	33.33	-267.87	42.88	17.64	-25.24	42.88	25	-17.88
1.5	0.083	1.5	301.2	33.33	-267.87	15.79	17.64	1.85	15.79	16.67	0.87
0.5	0.83	1.5	30.12	33.33	3.21	18.8	37.31	18.52	18.8	25	6.2
1.5	0.83	1.5	30.12	33.33	3.21	10.73	37.31	26.58	10.73	16.67	5.94

*Delays are given in minutes, rates in terms of cents/minute. Note that, across conditions, the signs of ΔSTR and ΔLTR are identical in the patch paradigm but not in the self-control paradigm—thus, STR maximization results in LTR maximization in the patch paradigm, but not necessarily in the self-control paradigm. Values are identical for both experiments*.

At the beginning of the session, participants were told that they would complete tasks that measure how people make decisions about different amounts of money available after different amounts of time, and that they would be paid the amounts they chose. Each participant completed four forced-choice demonstration trials (two each for the patch and self-control paradigms) and 20 experimental trials (10 for each paradigm). Experimental trials were always preceded by the paradigm-appropriate pair of forced-choice trials. In both experiments, E-prime 2.0 was used to present options to participants, collect their responses, and manage timing. See Figure S1 for example screenshots.

For Experiment 1, options were presented to participants on three computer monitors placed around a modular cubicle set-up (**Figure 1E**). Participants began trials at the point labeled “Home Station.” A button was pushed to begin a trial and participants waited for the duration of the ITI. Participants were then presented with one or two colored arrows.

For a Self-control trial, participants were simultaneously presented with a green and a blue arrow, one pointing right and one pointing left. If, for example, the left arrow was blue and represented *A_l_*, and the participant preferred that option, he or she clicked the blue arrow and then walked to the left (to the cubicle labeled “Left Station” in **Figure 1E**). Upon arrival, the participant pressed a button to begin *d_l_*. After the delay, the participant was presented a screen detailing the earnings for the trial. This screen included animated images of two U.S. $0.25 coins and was accompanied by a tone. The participant was then told to push a button to end the trial and return to the starting point to begin another trial. The side of the station associated with the larger-later reward was counter-balanced across experimental trials.

For a Patch trial, the participant was presented with a single arrow that was the color of the small reward (e.g., green) and that was either pointing left or right (counterbalanced across experimental trials). If the arrow was pointing right, for example, the participant clicked the arrow and then walked to his or her right (to the cubicle labeled “Right Station” in **Figure 1E**). The participant then pressed a button to begin *d_s_*. Following *d_s_*, the participant was presented with a reward screen, including an animation and tone, indicating the receipt of *A_s_*. After receiving the reward, the participant was presented with another screen indicating that he or she could either wait for the other reward (blue, in this example, worth $0.25, or *A_l_*–*A_s_*) or press a button to end the trial. If the participant ended the trial, he or she would then walk back to the “Home Station” and begin a new trial. For all trials, a running total of the amount participants had earned was kept at the bottom of each screen.

Two programming errors occurred in the implementation of this experiment. First, for conditions in the self-control paradigm in which participants should have received a 90-s ITI, participants received a 30-s ITI. Second, for conditions in which the larger-later reward was assigned the color green and when options were presented in the patch paradigm, participants were incorrectly directed to collect the green reward at the start of the trial instead of the blue reward. Supplemental analyses suggest that neither error was ultimately of consequence (see Supplemental Methods), but the results from Experiment 1 should be interpreted with these errors in mind.

The procedures for Experiment 2 were identical to those of Experiment 1, except that options were presented on a single computer screen and participants were not required to walk to receive reward. Additionally, all participants wore headphones so that they could not hear the reward tones generated by the other participants’ choices.

All procedures were approved by the University of Miami Institutional Review Board.

### STATISTICAL MODEL

Because each participant received 10 trials of each paradigm, data conformed to a two-level nested structure (trials nested within participants) in which paradigm was a within-subject factor and duration assignment a between-subject factor. Data were analyzed using a hierarchical linear model (HLM; Raudenbush and Bryk, 2001) in HLM version 7.01. This model was used to predict the log-odds that the large reward was chosen in a specific trial as a function of experimental treatment (i.e., ΔLTR) and the paradigm within which the choice was made (patch, coded as 1, or self-control, coded as 0). This analytic approach was used for both experiments.

The outcome variable was modeled as η*_ij_* = the log-odds that *A_l_* was chosen in trial *i* by person *j*, such that,

$$\eta_{ij}=\beta_{0j}+\beta_{1j}(Paradigm)+e_{ij}$$

$$\beta_{0j}=\gamma_{00}+\gamma_{01}(\Delta LTR)+u_{0j}$$

$$\beta_{1j}=\gamma_{10}+\gamma_{11}(\Delta LTR)+u_{1j}$$

where β*_0j_* and β*_1j_* are the intercept and slope coefficients, respectively, *u_0j_* and *u_1j_* are random effects representing the remaining variation between participants’ individual intercepts and slopes, and *e_ij_* represents residual within-person variation. The intercept (β*_0j_*) can be interpreted as the average log-odds for individual *j* that *A_l_* will be chosen when all other factors are zero (i.e., in the self-control paradigm). Differences between individuals’ intercepts that are due to differences in ΔLTR are modeled as the coefficient γ_01_. The slope (β*_1j_*) represents the change in the average log-odds for individual *j* when the trial occurs in the patch paradigm rather than in the self-control paradigm. Individual differences in this change that are due to differences in ΔLTR are modeled as the coefficient γ_11_. Missing data were handled using full information maximum likelihood, which provides unbiased parameter estimates and does not impute any data (Schafer and Graham, 2002). This modeling approach is preferable to approaches that assume fixed effects and require use of mean proportions of choices because it allows for generalizability beyond the samples used in our experiments and correctly models the outcome as a binomial process.

## RESULTS

**FIGURE 2:**
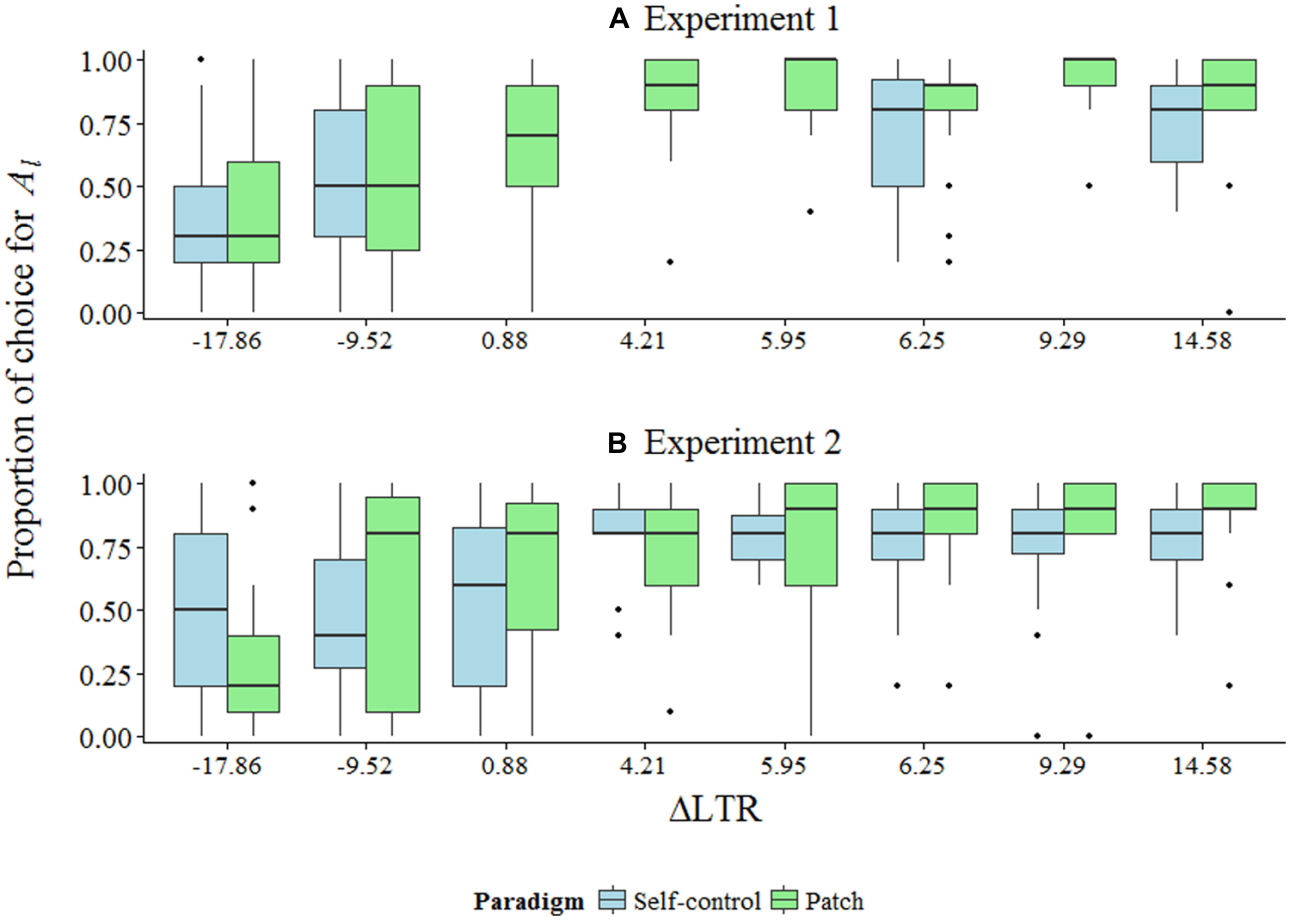
**(A,B)** Box plots of summary data for Experiments 1 and 2 given as proportion of trials during which *A_l_* was picked across ΔLTR conditions. Note that for Experiment 1, data for four conditions are missing for the self-control paradigm (see the text).

**FIGURE 3:**
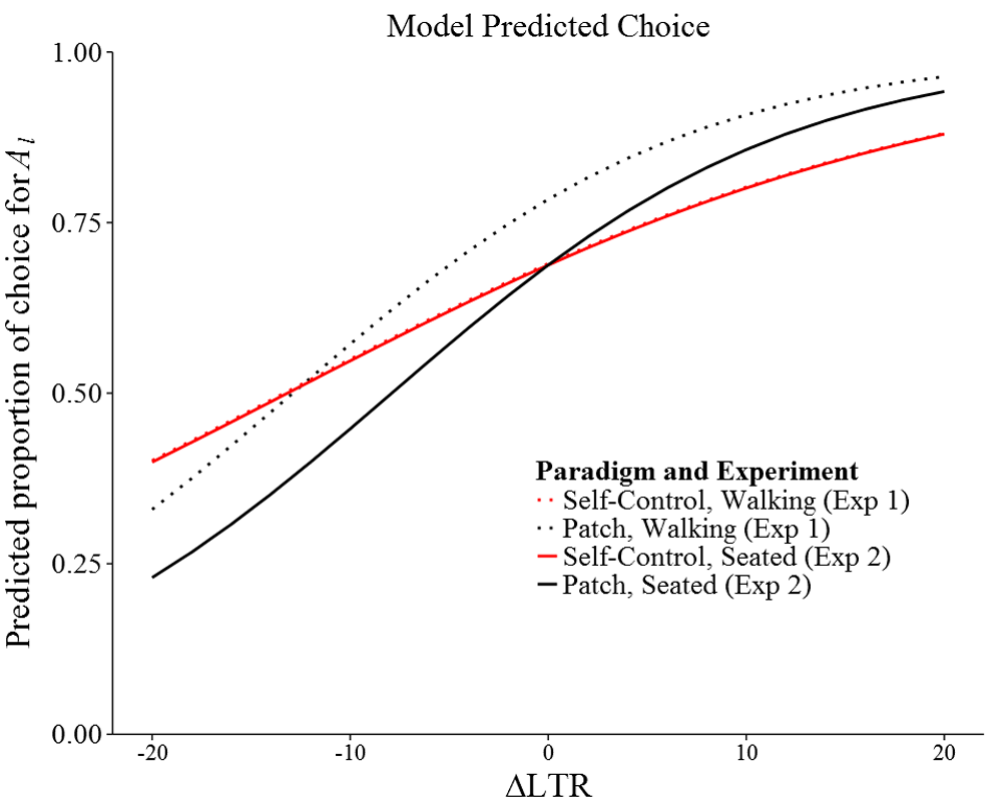
**Model predicted probability of picking *A_**l**_* by ΔLTR, paradigm, and experiment.** Not all ΔLTR values plotted were observed.

**Figures 2A,B** show summary data for both experiments. **Figure 3** shows model-predicted choices across a range of ΔLTR.

For Experiment 1, all terms in the model were statistically significant; for Experiment 2, all terms except for the main effect for paradigm were significant (**Table 2**). When all predictors in the model were zero—that is, when paradigm = 0 (i.e., a self-control trial) and ΔLTR = 0 (i.e., the LTRs of *A_l_* and *A_s_* are equal) participants were approximately twice as likely to choose *A_l_* as they were to choose *A_s_* in both experiments [Experiment 1 γ*_00_* = 0.80, odds ratio (OR) = 2.22; Experiment 2 γ*_00_* = 0.79, OR = 2.21]. In both experiments, this effect was modified by ΔLTR, such that an increase in ΔLTR by one cent/minute resulted in 6% (γ*_01_* = 0.06, OR = 1.06) increases in the likelihood of choosing *A_l_*. Thus, participants showed a noteworthy preference for *A_l_* when it gained them nothing, but participants were also sensitive to LTR in the self-control paradigm (as predicted).

**Table 2:** Results from both experiments based on a hierarchical linear model (HLM) predicting the log-odds of choosing *A_**l**_* as a function of ΔLTR and paradigm.

Experiments	Level 1		Level 2	Coefficient	Odds ratio	95% CI	
1	Intercept, *β_0_*		Intercept, *γ_00_*	0.80***	2.22	1.71–2.88	
			ΔLTR, *γ_01_*	0.06***	1.06	1.04–1.08	
	Paradigm, *β_1_*		Intercept, *γ_10_*	0.49***	1.64	1.23–2.19	
			ΔLTR, *γ_11_*	0.04**	1.04	1.01–1.06	
2	Intercept, *β_0_*		Intercept, *γ_00_*	0.79***	2.21	1.75–2.78	
			ΔLTR, *γ_01_*	0.06***	1.06	1.03–1.08	
	Paradigm, *β_1_*		Intercept, *γ_10_*	0.11	1.12	0.79–1.59	
			ΔLTR, *γ_11_*	0.04*	1.05	1.01–1.08	

*p < 0.05; **p < 0.01; ***p < 0.001.

In Experiment 1, a general tendency to choose *A_l_* rather than *A_s_* was observed when choices were made in the patch paradigm rather than the self-control paradigm and when ΔLTR = 0 (γ*_10_* = 0.49, OR = 1.64). This difference was not predicted, and it did not reach statistical significance for Experiment 2 (γ*_10_* = 0.11).

The primary prediction for both experiments (i.e., that the effect for paradigm would be modified by ΔLTR), was supported: when a choice was made in the patch paradigm, a one cent/minute increase in ΔLTR resulted in 4% (γ*_11_* = 0.04, OR 1.04) and 5% (γ*_11_* = 0.04, OR = 1.05) increases in the likelihood of choosing *A_l_* over *A_s_* in Experiments 1 and 2, respectively (i.e., the patch effect) over and above the effects obtained in the self-control paradigm.

The interactions between paradigm and ΔLTR (γ*_11_*) were decomposed by examining simple intercepts and simple slopes at each value of ΔLTR (**Table 3**; Preacher et al., 2006). In Experiment 1, we observed the patch effect for all conditions in which ΔLTR was positive (the difference in estimated odds ratios for picking *A_l_* instead of *A*_s_ ranged from 1.69 to 2.75; *p*s < 0.001). However, in both paradigms, choice for *A_s_* over *A_l_* when ΔLTR was negative did not reach statistical significance. It is worth noting that, although not statistically significant, the odds ratios for both the simple intercept and the simple slope when ΔLTR = -17.88 were in the predicted direction (**Table 3**). Additionally, at least half of the choices by at least half of the participants were for *A_s_* instead of *A_l_* when ΔLTR was negative (**Figure 1A**)—a distinctly different pattern of behavior than what was observed when ΔLTR was positive.

**Table 3:** The simple intercept represents a test of whether there is any preference for *A_**l**_* over *A_**s**_* in the self-control paradigm; the simple slope is the change in this preference in the patch paradigm as compared to the self-control paradigm.

		Simple intercepts		Simple slopes	
Experiments	ΔLTR	Log-odds (SE)	Odds ratio (*p*-value)	Log-odds (SE)	Odds ratio (*p*-value)
1	14.54	1.64 (0.20)	5.18 (*p* = 0.000)	1.01 (0.24)	2.75 (*p* = 0.000)
	9.27	1.34 (0.17)	3.81 (*p* = 0.000)	0.82 (0.19)	2.28 (*p* = 0.000)
	6.2	1.16 (0.15)	3.18 (*p* = 0.000)	0.71 (0.17)	2.04 (p = 0.000)
	5.94	1.14 (0.15)	3.13 (*p* = 0.000)	0.71 (0.17)	2.02 (*p* = 0.000)
	4.21	1.04 (0.14)	2.83 (*p* = 0.000)	0.64 (0.16)	1.90 (*p* = 0.000)
	0.87	0.85 (0.13)	2.33 (*p* = 0.000)	0.53 (0.15)	1.69 (*p* = 0.000)
	-9.55	0.24 (0.17)	1.27 (*p* = 0.149)	0.16 (0.18)	1.17 (*p* = 0.397)
	-17.88	-0.25 (0.23)	0.78 (*p* = 0.277)	-0.14 (0.26)	0.87 (*p* = 0.595)
2	14.54	1.60 (0.17)	4.93 (*p* = 0.000)	0.75 (0.27)	2.12 (*p* = 0.006)
	9.27	1.30 (0.13)	3.69 (*p* = 0.000)	0.52 (0.21)	1.68 (*p* = 0.014)
	6.2	1.13 (0.11)	3.11 (*p* = 0.000)	0.39 (0.19)	1.47 (*p* = 0.040)
	5.94	1.12 (0.11)	3.07 (*p* = 0.000)	0.37 (0.19)	1.45 (*p* = 0.046)
	4.21	1.02 (0.11)	2.79 (*p* = 0.000)	0.30 (0.18)	1.35 (*p* = 0.095)
	0.87	0.84 (0.11)	2.32 (*p* = 0.000)	0.15 (0.18)	1.16 (*p* = 0.398)
	-9.55	0.26 (0.20)	1.30 (*p* = 0.179)	-0.31 (0.27)	0.73 (*p* = 0.256)
	-17.88	-0.20 (0.29)	0.82 (*p* = 0.496)	-0.68 (0.39)	0.51 (*p* = 0.086)

For Experiment 2, we observed the patch effect when ΔLTR was 5.94 or above: estimated odds ratios for choices in the patch paradigm ranged from 1.45 to 2.12 higher than those in the self-control paradigm (*p*s < 0.046; **Table 3**). This same effect was nearly statistically significant for the condition where ΔLTR was 4.21 (OR = 1.35, *p* = 0.095). As in Experiment 1, participants were less successful at maximizing LTR when ΔLTR was negative compared to when it was positive. Specifically, when ΔLTR = -9.55, the odds ratios for both the simple intercept and the simple slope were not statistically significant (**Table 3**), and the majority of participants in the patch paradigm choose *A_l_* instead of *A_s_* the majority of the time (which, in both experiments, represents the only time participants failed to maximize LTR at least as well in the patch paradigm as in the self-control paradigm; **Figure 2B**). When ΔLTR = -17.88, the patch effect nearly reached statistical significance (OR = 0.50, *p* = 0.089).

## DISCUSSION

Our results contribute to a growing body of work that clearly demonstrates that intertemporal choice varies with the specifics of the procedure used to measure it. For example, when intertemporal choice is operationalized as a discount rate, the value of future reward apparently decreases faster for hypothetical consumable reward (e.g., food) than for hypothetical monetary reward (for a review, see Odum and Bauman, 2010). In addition, discount rates seem to be affected by a variety of experimental manipulations (Koffarnus et al., 2013). Intertemporal choice has also been found to be dependent on experimental context when measured as preference for the smaller-sooner reward (i.e., impulsive choice): for example, impulsive choice is higher for consumable rewards (e.g., juice; Logue and King, 1991), in the presence of visual food cues (Forzano and Corry, 1998), and when distractions are present during choice (Kirk and Logue, 1996). For our results, the most relevant of these types of findings is that in experiments most similar to ours, participants apparently adopt LTR-maximizing strategies, even when such strategies prescribe impulsive choice (e.g., Logue et al., 1986; Flora and Pavlik, 1992; Ito and Nakamura, 1998). We replicated this finding in our two experiments when ΔLTR was positive (the simple intercepts in **Table 3**), and found that, although not statistically significant, at least half of the subjects chose the smaller-sooner option on at least half of the trials when ΔLTR was negative (**Figure 1A,B**). Additionally, we found that participants were generally more successful at maximizing LTR when options were presented in the patch paradigm rather than in the self-control paradigm (i.e., the patch effect): across the two experiments, the likelihood of choosing the LTR maximizing option was statistically significantly increased in 10 of 16 cases (*p*s < 0.05), and nearly significantly increased in two additional cases (*p*s < 0.10; see the simple slopes in **Table 3**), one of which represented a LTR-appropriate reduction in preference for the larger-later reward (i.e., LTR-maximizing, but impulsive choice). Overall, our results (**Figure 3**) were broadly consistent with the choices by blue jays reported by Stephens and Anderson (2001).

The finding that participants apparently adopt LTR-maximizing strategies in some versions of the self-control paradigm but not others, and that the ability to maximize LTR is improved if options are presented as a foraging-style leave/stay choice, suggests that choice in different contexts may be produced by different cognitive mechanisms. Other findings support this conclusion, as well: the same person’s discount rate seems to depend on the version of the self-control paradigm experienced (e.g., questionnaire procedures or more operant-style ones; Navarick, 2004), and discount rates across procedures are not necessarily correlated (Lane et al., 2003a,b). Moreover, participants clearly do not use LTR-maximizing strategies when faced with some popular questionnaire versions of the self-control paradigm, such as the monetary choice questionnaire (MCQ; Kirby and Maraković, 1996)—if they did, choice would exclusively favor the smaller-sooner option, and discount rates would be much higher than are typically reported (e.g., Carter et al., 2012).

It is not clear what determines the choice strategy or mechanism in use during different versions of the self-control paradigm, or even why discounting rate can be modified by so many factors (Koffarnus et al., 2013); however, it is important to note that even the most successful delay discounting models, such as the hyperbolic model (Mazur, 1987), do not provide an explanation for such context-dependence (van den Bos and McClure, 2013). These models are also limited in that they almost uniformly describe discounted value as a function of only the prereinforcer delay. This is problematic for cases where choice changes in the face of manipulations of postreinforcer delays, since standard discounting models can only account for such variation through discounting parameters. For example, Flora and Pavlik (1992) presented two groups of subjects with a choice between an immediately available 4 points, worth one cent each, and 10 points after 15 s. The options presented to the groups differed only in that one group was exposed to a postreinforcer delay of 15 s following receipt of the smaller-sooner reward. In terms of LTR, the addition of the postreinforcer delay switched the sign of ΔLTR for these conditions from negative to positive, and choice of the larger-later reward between the two groups tracked this switch: in the absence of the postreinfrocer delay, only one subject chose the larger-later option, and only on a third of trials, whereas in the presence of the postreinforcer delay, all subjects chose the larger-later option on the vast majority of trials. Fitting a standard discounting model to these data would produce the result that subjects in the two groups discounted future reward at very different rates—that is, participants in the postreinforcer delay condition would have appeared to discount delayed reward less steeply than those in the condition lacking the postreinforcer delay. Although possible, such an explanation is unlikely given that subjects were taken from the same population and randomly assigned to groups.

Standard discounting models fail to reasonably account for choice in our experiments for similar reasons. In the self-control paradigm, for conditions where ΔLTR = -17.88, -9.55, 0.87, or 4.21, the *k* values from the hyperbolic model at indifference between *A_l_* and *A_s_* would be *k* = 0.0125, 0.02, 0.0125, and 0.02, respectively. In these conditions, preference for *A_l_* over *A_s_* suggests that participants’ *k* values are *less* than the *k* values at indifference, and for conditions where ΔLTR = 0.87 or 4.21, this seemed to be the case (**Figure 2B**). However, if participants’ *k* values were that small across groups (which, given random sampling, they likely were), then participants also should have shown an equivalent preference for *A_l_* in conditions where ΔLTR = -9.55 or -17.88. Instead, when ΔLTR changed sign from ΔLTR = 0.87 and 4.21 to ΔLTR = -17.88 and -9.55, which was achieved by manipulating ITI, participants showed a decrease in choice for *A_l_* to the point where neither *A_l_* nor *A_s_* was preferred (see the simple intercepts in **Table 3**; **Figures 2A,B**). If one is willing to assume comparable *k* values across groups, then the hyperbolic model does not account for our findings.

Critically, the patch effect is predicted by standard discounting models in some cases. For example, in our experiment, for conditions in ΔLTR = 0.87 or 4.21, if participants’ *k* values were *greater* than *k* = 0.0125 or 0.02, respectively, one would predict preference for *A_s_* in the self-control paradigm, but *A_l_* in the patch paradigm. However, as mentioned, the data strongly suggest that *k* values for these participants would be less than *k* = 0.02 (e.g., choice in the self-control paradigm when ΔLTR = 4.21 in Experiment 2 strongly favors *A_l_*, implying *k* < 0.02; **Figure 2B**), and therefore not large enough to predict the patch effect. Of course, the current experiments were not designed to assess individual discounting parameters, so the appropriateness of standard discounting models for our data cannot be truly tested. Considering that variation in discounting parameters can predict the patch effect, future experiments should be carefully designed to rule out such a possibility.

Given our data, we can only speculate on the mechanism(s) underlying the patch effect. One possibility is that the patch effect is due to an improvement in participants’ ability to determine which option maximizes LTR. Maximizing LTR is critically dependent on being able to discriminate the LTRs of the options, and the further ΔLTR is from zero, the more obvious it is which choice maximizes LTR. In line with this interpretation, we found that the probability of choosing *A_l_* was positively related to ΔLTR: γ_01_ for both experiments; **Table 2** (see Shenhav et al., 2014 for a similar discussion of human choice behavior on a foraging-type task). The positive relationships between the probability of picking *A_l_* and ΔLTR increased during choice in the patch paradigm compared to in the self-control paradigm (γ_11_ for both experiments; **Table 2**), which is consistent with the interpretation that the patch effect is due to an increased ability to discriminate between the LTRs of the two options.

One possibility for why discriminability might be improved in the patch paradigm is that participants could be better able to understand that they are facing a *sequence* of choices in the patch paradigm, and so are more likely to consider the options in terms of rates, rather than, say, simply the amount of reward associated with an option. Given that one option in the patch paradigm explicitly involves moving on to the next trial (i.e., the leave option; **Figure 1D**), whereas no option in the self-control paradigm relates to any trial beyond the current one (**Figure 1C**), we find this explanation to be particularly promising.

Future work can improve upon the experiments we report here in several ways. First, one potential explanation for the patch effect is that the pattern of reward receipt differs in the two paradigms: in the patch paradigm, there are two deliveries for the stay option, one of which is delivered right before the choice point (**Figure 1B**), compared to only one delivery in the self-control paradigm, always following the end of the prereinforcer delay (**Figure 1A**). This might increase choice for the stay option if, for example, reward delivery itself was rewarding above and beyond the value of the reward received (e.g., by virtue of interrupting a boring delay period). Stephens and Dunlap (2009) examined the issue of multiple deliveries with blue jays by splitting the delivery of the larger-later reward in the self-control paradigm up so that it exactly matched reward delivery of the stay option in the patch paradigm. Interestingly, this manipulation did increase choice for the larger-later reward in the modified self-control paradigm as compared to the patch paradigm and the standard self-control paradigm, but only when such choice was inconsistent with LTR (i.e., a non-optimal preference for the larger-later option). This is in line with the argument that subjects are in fact motivated by multiple deliveries, but the same blue jays preferred the leave option—the option with only one delivery—to the stay option in the patch paradigm when leaving was the LTR-maximizing option. These findings, in conjunction with other data from blue jays showing LTR-consistent preference for leaving in the patch paradigm (e.g., Stephens and Anderson, 2001), indicate that although a reward split into multiple deliveries might be preferred to a reward delivered all at one time, such preference is not enough to fully explain the patch effect. Of course, the same cannot be definitively said for our human participants, given that leaving in the patch paradigm was never statistically significantly preferred to staying (although it nearly was for Experiment 2 when ΔLTR = -17.88, and the strong preference for staying that was observed when ΔLTR was positive did reduce to at least indifference when ΔLTR was negative and leaving was the LTR-consistent option; **Table 3**). A direct test of the multiple deliveries hypothesis using human subjects would be useful.

It would also be beneficial to conduct future experiments in which various properties of the reward are manipulated. For example, given that human subjects seem to behave less in line with LTR-maximization when reward in the self-control paradigm is consumable (e.g., Logue and King, 1991), testing the patch effect for such reward would be useful. Additionally, the validity of the patch paradigm as an analog for patch-foraging could be improved by setting the reward to more obviously diminish over time. Although it would make direct comparison to the classic self-control paradigm more difficult, the reward sequence for the stay option could be split across more delivery events in such a way that each subsequent reward was slightly smaller than the last. Previous work with human subjects suggests that choice in such a context would approximately track LTR-maximization (Hackenberg and Axtell, 1993).

Furthermore, it might also be productive to manipulate the information given to participants during choice. For example, it may be the case that providing information about the total reward earned biases behavior over time, so preference could be manipulated by removing this information. Additionally, giving explicit cues about the timing of non-prereinforcer delays, such as the ITI, should improve choice (Pearson et al., 2010; Blanchard et al., 2013). Manipulating such factors might help to flesh out exactly why the patch effect exists—for example, it might be the case that the ITI is more salient in the patch paradigm, thereby making the LTR-maximizing option more obvious.

Moreover, a better understanding of decision-making in the patch paradigm could be obtained by continuing to examine how choice changes when options are spatially separate, as in Experiment 1. Relative to choice in Experiment 2, our subjects seemed to prefer the stay option in the patch paradigm when leaving required physically moving away from a station (**Figure 3**), even to the detriment of maximizing LTR. Such “over-staying” is frequently observed in patch foraging (Nonacs, 2001), and whether it is due to some form of discounting by physical effort or adherence to a type of “win-stay” strategy, it seems to be a regular feature of decision-making during patch foraging. Unfortunately, Experiment 1 was limited by two programming errors, and although statistically accounting for the errors suggested that they were of little consequence (see Supplemental Materials), it would be beneficial to repeat Experiment 1 in the future so as to have a solid foundation from which to study the effect of physical movement on choice in the patch paradigm.

Finally, future work could also expand on the present design by testing alternative criteria for ending sessions. For example, in the current work, it is possible that participants were aware of the fact that the end of a session was trial-based rather than time-based (despite being told otherwise). If so, the average bias toward picking *A_l_* (γ*_00_* for both experiments in **Table 2**) might have represented a strategy based on optimizing the total amount of reward received over the number of trials allowed, rather than the rate of reward calculated based on the time in a trial. However, the fact that choice was sensitive to ΔLTR at least indicates that this strategy was not adopted by all participants or at all times. Ruling this possibility out would be useful for future work using either paradigm.

### COMPARING LEAVE/STAY CHOICES TO LARGER-LATER/SMALLER-SOONER CHOICES

Based on findings such as ours (e.g., Stephens and Anderson, 2001) it seems clear that any model or hypothesis about intertemporal choice ought to address the structure of the choice problem being faced. One might argue that considering instances of choice that do not follow the larger-later/smaller-sooner structure is unnecessary if the primary goal is understanding—and modifying—real-world examples of human behavior in which future consequences seem to be devalued (e.g., addiction, gambling, obesity). Presumably, such an argument would rest in part on the logic that these real-world examples seem to be larger-later/smaller-sooner-type choices (e.g., a choice between the short-term benefits of a drink versus the long-term benefits of abstinence). However, we think these examples can be profitably seen as following a leave/stay structure instead. For example, in the case where a person addicted to alcohol is faced with the decision to drink or to abstain, the choice to drink can be thought of as a choice to engage in a stay strategy: engaging in a behavior to receive some estimated payoff (i.e., the subjective value of the drink). On the other hand, the choice to abstain can be thought of as enacting a leave strategy: continuing to search out other options based on the expectation of the types of payoffs that might be encountered elsewhere (e.g., any potential benefits to personal or professional relationships that might otherwise be put at risk through drinking). Importantly, if this person were to choose to drink when the option became available (the stay strategy), he or she could then change his or her mind and stop drinking (the leave strategy)—perhaps as the expected value of other possible outcomes in the environment increased. Thus, compared to the larger-later/smaller-sooner conceptualization of this same example, the leave/stay formalization models the mutual exclusivity of the options more realistically—that is, one cannot drink and abstain at the same time, but a single choice to drink in the moment does not forever remove the option of abstaining in the future, as is suggested by the mutual exclusivity of the smaller-sooner and larger-later reward in the self-control paradigm.

Beyond considering real-world examples of impulsivity, a host of other intertemporal choice problems can also be seen as taking a leave/stay structure. For example, various authors have applied essentially this same framework to decisions about when to halt visual search (Wolfe, 2013), how to manage time while gathering information (Pirolli, 2005), when to give up during problem solving (Payne and Duggan, 2011), when to engage in a task requiring executive function (Kurzban et al., 2013), and when to stop spending time doing an “internal search,” as in recalling information from memory (Wilke et al., 2009; Hills et al., 2012). Many other problems neatly fall into this framework, although, to our knowledge, have not been studied as such (e.g., decisions about when to switch careers or leave a romantic relationship, about when to stop preparing for an exam, even decisions about when to stop collecting data during experimental research). Thus, given the apparent ubiquity of leave/stay problems, we believe that any fully developed theory of intertemporal choice, including theories of how to modify intertemporal choice, must account for how decisions are made in this context.

## CONCLUSION

We found that human participants’ choices were more optimal—in the sense that they were more in line with LTR-maximization—in the patch paradigm as compared to in the self-control paradigm in 12 of 16 conditions across two experiments (with varying levels of statistical significance: *p*s from <0.10 to <0.001; **Table 3**). Furthermore, choice in the patch paradigm maximized LTR at least as well as, but in most cases better than, choice in the self-control paradigm in all but one condition (**Figures 2A,B**).

A fully developed account of intertemporal choice must consider the ways in which choice changes based on how options are presented (e.g., as a larger-later/smaller-sooner choice or a leave/stay choice). Our results add to a body of work that strongly indicates that current discounting models are very limited descriptions of intertemporal choice, and that researchers would do well to consider choice paradigms beyond the self-control paradigm. These points are important to consider for research on real-world examples of impulsivity, since they suggest that efforts to understand or modify such behaviors will depend crucially on the ways in which they are operationalized. Specifically, if delay discounting behavior is indeed a trans-disease process (Bickel et al., 2012), is the same true of all intertemporal choice behavior? Or is how an individual handles a larger-later/smaller-sooner choice the only useful predictor of disease outcomes? Investigating such questions will ultimately be critical to a full understanding of intertemporal choice, both in the laboratory and in the real world.

## Conflict of Interest Statement

The authors declare that the research was conducted in the absence of any commercial or financial relationships that could be construed as a potential conflict of interest.
