# Supplementary material for: Reassessing intertemporal choice: human decision-making is more optimal in a foraging task than in a self-control task
Source: Front Psychol. 2015 Feb 6;6:95. doi: 10.3389/fpsyg.2015.00095 (PMC4344112; doi:10.3389/fpsyg.2015.00095)
Supplement: Supplementary file 1 [file Presentation_1.PDF]

Supplemental Methods

Figure S1 depicts screenshot examples of trials in both paradigms.

Two programming errors occurred while designing Experiment 1 (both were corrected for Experiment 2). First, for timing conditions in the self-control paradigm in which participants should have received a 90-second ITI, participants only received a 30-second ITI. We handled this error by coding choice data for trials in the self-control paradigm for these participants (n = 106) as missing and running the model with full information maximum likelihood (FIML) estimation, which provides unbiased parameter estimates and does not impute any values. We also applied the same statistical analysis described in the main text to the data from Experiment 1 without accounting for missing data (Table S1). The results from this analysis are essentially the same as those that were found when applying FIML. Given that the results were the same whether FIML was used or not, and that the parameter estimates in the model for Experiment 1 are nearly identical to those for Experiment 2, we are confident that the results obtained are not significantly biased due to this programming error.

Second, for conditions in which the large reward was assigned the color green and when options were presented in the patch paradigm, participants were incorrectly directed to collect the green reward at the start of the trial (recall that participants should have been directed to first collect the small reward, which in this case, would have been the blue reward). Upon arrival at the correct station, participants were then correctly instructed to push a button for the blue reward. The rest of the trial proceeded correctly. The training trials that preceded these flawed trials did not include this error. We handled this error by running an additional model to test for the interaction between paradigm and color assignment and to control for the possible influence of color.

As in the primary analysis, data were analyzed using a Hierarchical Linear Model (HLM) in HLM version 7.01 (Raudenbush & Bryk, 2001).

A model was run to account for the color programming error in Experiment 1. This model was used to predict the likelihood that A_l was chosen in a specific trial as a function of the experimental treatment that the participant received (change in long-term rate, or ΔLTR, which takes the form of change in cents/minute gained when A_l is chosen over A_s), the paradigm (patch, coded as 1, or self-control, coded as 0) within which the choice was made, and the color (either blue, coded as 1, or green, coded as 0) that was assigned to the large reward. This model took the form

$$\eta_{ij} = \beta_{0j} + \beta_{1j}(\text{Paradigm}) + e_{ij},$$

$$\beta_0 = \gamma_{00} + \gamma_{01}(\Delta\text{LTR}) + \gamma_{02}(\text{Color}) + \mu_{0j},$$

$$\beta_1 = \gamma_{10} + \gamma_{11}(\Delta\text{LTR}) + \gamma_{12}(\text{Color}) + \mu_{1j}.$$

Both terms for color (γ_{02} and γ_{12}) were statistically non-significant (suggesting that the color that was assigned to the large reward did not influence preferences), whereas both terms for ΔLTR (γ_{01} and γ_{11}), and the intercept (γ_{00}) were statistically significant, as was the case in the primary analysis reported in the primary text. One difference between this model and the model in the primary analysis was that in the presence of the terms for color, the slope for paradigm (γ_{01}) became (marginally) statistically non-significant. This finding indicates that there was no longer an influence on choice due simply to the type of paradigm a choice was made in, but rather, the influence of paradigm was dependent on ΔLTR (as indicated by the statistically significant term, γ_{11}). This result is similar to the findings of experiment two. This change in results has little bearing on the overall conclusions derived from the primary analysis, since our

hypothesis was specifically about the interaction between paradigm type and ΔLTR; however, it does make interpretation of the significant main effect for paradigm in Experiment 1 somewhat fraught: This effect may be due to the methodological specifics of Experiment 1, or it may be due to the programming error. See Table S2 for results.

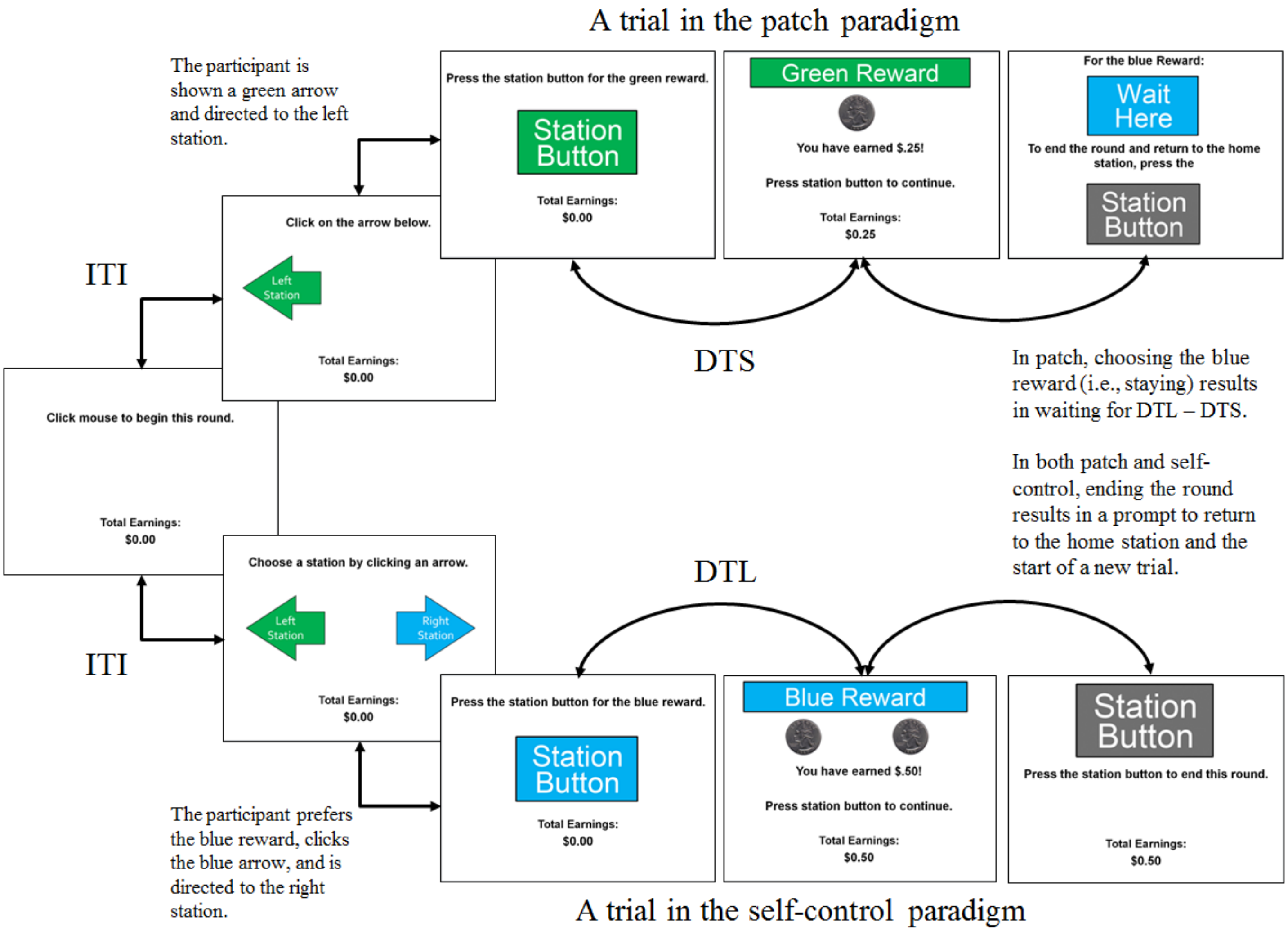

Figure S1. Screenshots for portions of trials in the patch and self-control paradigms in experiment one. The above design was different for experiment two in that the blue and green arrows were changed to squares, there was no mention of stations, and the option presented as "wait here" at the choice point in the patch paradigm simply read "wait."

		Unit-specific coefficient	*p*-value	Odds ratio	95% CI for odds ratio
Intercept, β_0	Intercept, γ_{00}	0.77	.000	2.16	1.66 to 2.81
	ΔLTR, γ_{01}	0.06	.000	1.06	1.04 to 1.08
Paradigm, β_1	Intercept, γ_{10}	0.44	.004	1.55	1.16. to 2.01
	ΔLTR, γ_{11}	0.03	.017	1.03	1.01 to 1.06

Table S1. Results of an HLM predicting the log-odds of choosing A_l in a trial as a function of ΔLTR and the paradigm within which the choice was made. The model was run without full information maximum likelihood estimation.

		Unit-specific coefficient	*p*-value	Odds ratio	95% CI for odds ratio
Intercept, β_0	Intercept, γ_{00}	0.66	.000	1.94	1.31 to 2.88
	ΔLTR, γ_{01}	0.06	.000	1.06	1.04 to 1.08
	Color, γ_{02}	0.26	.320	1.30	0.77 to 2.20
Paradigm, β_1	Intercept, γ_{10}	0.39	.059	1.90	0.99 to 2.21
	ΔLTR, γ_{11}	0.04	.005	1.04	1.01 to 1.06
	Color, γ_{12}	0.21	.471	1.23	0.70 to 2.19

Table S2. Results of an HLM predicting the log-odds of choosing A_l in a trial as a function of ΔLTR, the paradigm within which the choice was made, and the color used to represent A_l.
